# Acute Pancreatitis Complicated by Stress Cardiomyopathy With Persistent Apical Akinesis: A Case Report and Literature Review

**DOI:** 10.1177/2324709619893197

**Published:** 2019-12-10

**Authors:** Temidayo Abe, Melvin Simien, Hayes Dolphurs

**Affiliations:** 1 Morehouse School of Medicine, Atlanta, GA, USA

**Keywords:** stress cardiomyopathy, pancreatitis, takotsubo

## Abstract

Takotsubo cardiomyopathy or stress cardiomyopathy is a transient reversible cardiomyopathy characterized by regional wall motion abnormalities that usually extend beyond a single epicardial vascular distribution. It is often precipitated by acute physical or emotional stressors. In this article, we present the case of a postmenopausal woman who was admitted for management of acute pancreatitis. On the second day of admission, she developed shortness of breath and electrocardiographic abnormalities. A transthoracic echocardiogram revealed left ventricular systolic dysfunction and apical akinesis, and coronary angiography revealed normal coronary arteries. She was diagnosed with takotsubo cardiomyopathy triggered by acute pancreatitis and started on guideline-directed heart failure medications. A follow-up echocardiogram 4 months later revealed persistent systolic dysfunction and apical akinesis.

## Introduction

Takotsubo cardiomyopathy (TCM) or stress cardiomyopathy is a transient reversible cardiomyopathy characterized by regional wall motion abnormalities that usually extend beyond a single epicardial vascular distribution in the setting of emotional or physical stress.^1,2^ The incidence of TCM has increased in recent years due to the availability of early invasive coronary angiography and increased awareness.^3^ In this case report, acute pancreatitis was the precipitating factor. While acute pancreatitis remains the leading gastrointestinal cause of hospitalization in the United States, the likelihood of developing secondary TCM is very rare.^3^ To promote better understanding, we reviewed all cases of pancreatitis-induced TCM in the current literature.

## Case Report

A 57-year-old African American female with a history of alcohol abuse and diabetes mellitus presented to the emergency department with a 2-day history of severe diffuse abdominal pain with radiation to the back. Associated symptoms included nausea and vomiting. She consumed 5 bottles of beer daily with the most recent alcohol intake 2 days prior to presentation. Vital signs on presentation were blood pressure 123/90 mm Hg, pulse 125 beats/minute, respiratory rate 17 breaths/minute, and temperature 36.8°C. Physical examination was significant for a mildly tender abdomen. Laboratory findings revealed leukocytosis of 14 600/mm^3^ and lipase of 882 U/L (normal = 16-62 U/L). Computed tomographic imaging of the abdomen with and without contrast revealed peripancreatic fat stranding suggestive of acute interstitial pancreatitis. The patient was admitted for intravenous fluid resuscitation and pain management.

**Figure 1.**
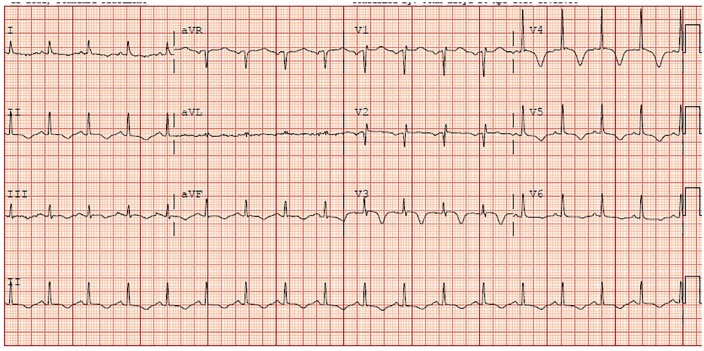
Electrocardiogram with T-wave inversion in V3-V5, II, III, and aVF.

**Figure 2.**
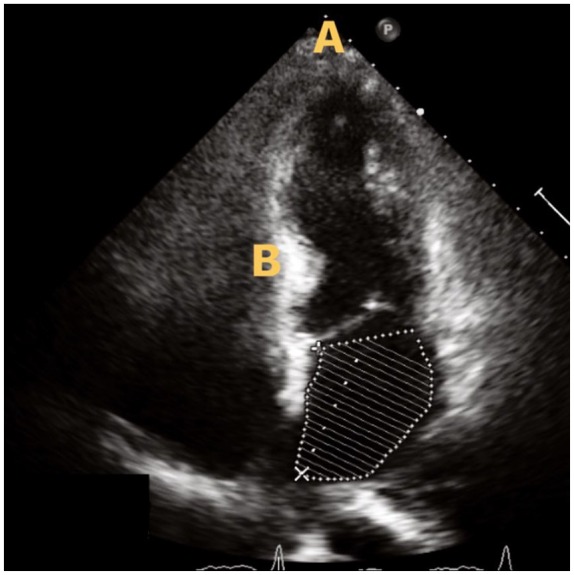
Echocardiogram reveals apical akinesis (A) and basal hyperkinesis (B) consistent with takotsubo cardiomyopathy.

On day 2 of admission, the patient became dyspneic and hypoxemic (digital pulse oximetry 82%) on room air. Chest radiography showed pulmonary edema, and abdominal ultrasound revealed a dilated inferior vena cava. Troponin I 0.97 ng/mL (normal <0.03 ng/mL) and brain natriuretic peptide 1627 pg/mL (normal <100 pg/mL) levels were elevated. A 12-lead electrocardiogram (ECG; Figure 1) obtained revealed diffuse ischemic T-wave inversion. The patient was aggressively diuresed with significant improvement in respiratory status. Transthoracic echocardiography revealed a left ventricular ejection fraction of 40% with basal segment hyperkinesis but apical akinesis consistent with stress-induced cardiomyopathy (Figure 2). Coronary angiography revealed normal coronary vessels.

The patient’s abdominal pain resolved, and there were no further complications from her pancreatitis. She was started on an angiotensin converting enzyme (ACE) inhibitor and a β-blocker and discharged to home in stable condition. A repeat transthoracic echocardiography obtained 4 months later revealed unchanged left ventricular ejection fraction and persistent apical akinesis in the setting of persistent alcohol use. The ACE inhibitor and β-blocker were continued, and she was counselled on alcohol cessation.

## Discussion

Takotsubo cardiomyopathy is a clinical syndrome characterized by severe ventricular dysfunction in the absence of obstructive coronary artery disease with regional wall motion abnormalities that usually extend beyond a single epicardial vascular distribution.^2,4^ Since its discovery in the 1990s, it has been increasingly recognized in recent years.^4,5^ On presentation, clinical signs and symptoms are usually consistent with acute coronary syndrome. Current diagnostic criteria include the following: (1) transient left ventricular dysfunction (hypokinesia, akinesia, or dyskinesia) presenting as apical ballooning or midventricular, basal, or focal wall motion abnormalities; (2) usually an emotional, physical, or combined trigger; (3) new ECG abnormalities are present (ST-segment elevation, ST-segment depression, T-wave inversion, and QTc prolongation); (4) levels of cardiac biomarkers (eg, troponin) are moderately elevated and significant elevation of brain natriuretic peptide is common; (5) absence of significant coronary artery disease; and (6) no evidence of infectious myocarditis.^6^ Initially considered a benign disease, recent studies have demonstrated mortality and morbidity in patients with TCM.^1^

Although the exact pathogenesis remains unclear, it is proposed to be secondary to exaggerated myocardial catecholamine exposure, which may induce myocardial damage from direct toxic effects or indirectly via microvascular spasm in predisposed patients due to genetic mutation, underlying endothelial dysfunction or reduced in estrogen levels.^1,7^ Also, regional differences in myocardial β-adrenergic receptors densities coupled with a downregulation of the receptors by exaggerated catecholamine exposure are thought to play a role.^7^ It is predominantly seen in postmenopausal women in a setting of an emotional or physical stressor (ie, acute lung diseases and central nervous system disorders).^8^ Notably, patients who developed TCM as a result of physical stressors tend to have a poorer prognosis with one study reporting in-hospital mortality rate of 20.9% compared with 2.6% in those with an emotional stressor.^9^

**Figure 3.**
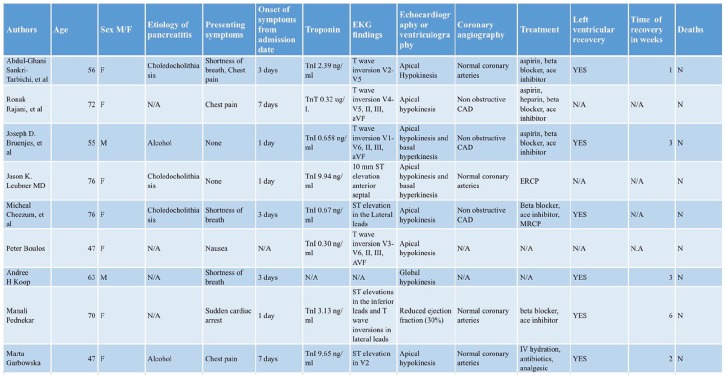
Cases of pancreatitis induced takotsubo reported in the literature. References [10-16]. Abbreviations: M, male; F, female; N/A, not available or provided by the authors; TnI, troponin I; TnT, troponin T; ERCP, endoscopic retrograde cholangiopancreatography; % MRCP, magnetic resonance cholangiopancreatography %.

Takotsubo cardiomyopathy in association with acute pancreatitis, as observed in our patient, has rarely been reported. To our knowledge, only 9 cases have been previously reported (Figure 3).^10-16^ Most patients were female (7/9) and >55 years of age (7/9). All patients presented within a week of pancreatitis onset, often with shortness of breath and chest pain; however, one patient presented with sudden cardiac death. Troponin was elevated in all patients, ranging from TnI 0.32 ng/mL to 9.94 ng/mL. As with our case, ECG findings were mostly T-wave inversion, especially in the anterior leads (n = 4/7). Most patients had apical akinesis with basal hyperkinesis (n = 8/9). Most patients were reportedly treated with heart failure medications (n = 5/7), and unlike our case, ventricular function normalized within 6 weeks for all patients with follow-up imaging studies (n = 6/6).

Complete recovery of left ventricular systolic dysfunction and wall motions abnormalities is expected within 2 months of onset.^17-19^ The unique feature of our case is persistent left ventricular apical akinesis at 4 months follow-up despite treatment with an ACE inhibitor and a β-blocker. We suspect that persistent alcohol abuse might be contributing to the delayed left ventricular recovery in our patient; however, it has not been reported in the literature. More recent studies evaluating left ventricular recovery pattern with 2-dimensional speckle-tracking echocardiography in patients with TCM suggests that left ventricular recovery might not be complete as previously suggested.^20,21^

## Conclusion

Takotsubo cardiomyopathy should be considered in patients with pancreatitis who develop clinical signs and symptoms suggestive of acute coronary syndrome. Despite our increased understanding of the clinical implications of TCM, more studies are needed with regard to long-term follow-up and recovery patterns.
